# Knowledge and perceptions of food sustainability in a Spanish university population

**DOI:** 10.3389/fnut.2022.970923

**Published:** 2022-11-29

**Authors:** M. Clara de Moraes Prata Gaspar, Ricard Celorio-Sardà, Oriol Comas-Basté, M. Luz Latorre-Moratalla, Mari Aguilera, Gustavo A. Llorente-Cabrera, Montserrat Puig-Llobet, M. Carmen Vidal-Carou

**Affiliations:** ^1^Departament d’Antropologia Social, Facultat de Geografia i Història, Universitat de Barcelona (UB), Barcelona, Spain; ^2^Institut de Recerca en Nutrició i Seguretat Alimentària (INSA-UB), Universitat de Barcelona, Santa Coloma de Gramenet, Spain; ^3^Departament de Nutrició, Ciències de l’Alimentació i Gastronomia, Facultat de Farmàcia i Ciències de l’Alimentació, Campus de l’Alimentació de Torribera, Universitat de Barcelona (UB), Santa Coloma de Gramenet, Spain; ^4^Xarxa d’Innovació Alimentària (XIA), Barcelona, Spain; ^5^Departament de Cognició, Desenvolupament i Psicologia de l’Educació, Secció Cognició, Facultat de Psicologia, Universitat de Barcelona (UB), Barcelona, Spain; ^6^Institut de Neurociències (UBNeuro), Universitat de Barcelona (UB), Barcelona, Spain; ^7^NeuroDevelop eHealth Lab, eHealth Center, Universitat Oberta de Catalunya (UOC), Barcelona, Spain; ^8^Departament de Biologia Evolutiva, Ecologia i Ciències Ambientals, Facultat de Biologia, Universitat de Barcelona (UB), Barcelona, Spain; ^9^Institut de Recerca de la Biodiversitat (IRBio), Universitat de Barcelona (UB), Barcelona, Spain; ^10^Departament d’Infermeria de Salut Pública, Salut Mental i Maternoinfantil, Facultat de Medicina i Ciències de la Salut, Campus Bellvitge, L’Hospitalet de Llobregat, Spain

**Keywords:** sustainability, food, perception, knowledge, environmental impact, university population

## Abstract

In 2015, the United Nations adopted the 2030 Agenda for Sustainable Development, with 17 Sustainable Development Goals (SDGs) at its core. Besides tackling climate change and the fight to reduce inequality, the SDG number 12 is specifically focused to develop strategies toward food sustainability. The aim of this study, aligned with SDG number 12, was to analyze the level of knowledge and perceptions of food sustainability in a university community from Spain. A descriptive cross-sectional study, based on an online questionnaire, was carried out between July and November 2021 with convenience sampling. The survey included 28 items and was distributed among students, teachers, researchers and administrative staff from a Spanish university. A total of 1,220 participants completed the survey. 70.4% of the respondents heard about the environmental impact of food and more than 50% were aware of the existence of the SDGs. The different aspects related to diet that concerned them the most were food waste, plastic usage, and environmental impact. They reported that a sustainable diet should be mainly based on local and seasonal products and with a low environmental impact as well as no or the minimum food waste. When asked if they were following a sustainable diet, 77% answered affirmatively. Moreover, the food groups more involved in a sustainable diet should be vegetables and fruits, olive oil, legumes, and whole grains. Regarding food waste, 60% of the surveyed population claimed to generate it at home, with the use of leftovers and planning shopping and meals being some of the most important domestic actions to avoid it. Further initiatives must be implemented to increase the level of knowledge as well as to raise the awareness on the importance to translate it into individual and collective actions that allow a shift toward more sustainable practices.

## Introduction

The modern food system faces an unprecedented challenge: on the one hand, to manage the environmental and socioeconomic consequences of the industrial production model, and on the other, to produce affordable and nutritious food in adequate quantities in a context of population growth in a sustainable and resilient manner, reducing environmental impacts and the overexploitation of natural resources (1–3). In this scenario, sustainability has become a key concept of new strategies promoting a global transformation of the current food system (4). Sustainability is a complex multidimensional notion that encompasses the simultaneous fulfillment of different objectives with productive, ecological, temporal, economic and socio-cultural dimensions (5, 6).

In 2015, the General Assembly of the United Nations adopted the 2030 Agenda for Sustainable Development, with 17 Sustainable Development Goals (SDGs) at its core. These SDGs are an urgent call for action to all countries in a global partnership to improve the future of people and the planet. Besides tackling climate change and inequality, the SDGs are also focused on developing strategies to foster a healthy and sustainable diet. According to Lang (7), defining what constitutes a sustainable diet is a major challenge because it is not only a matter of reconciling discourses of public health with those of ecology, but also includes the economic and cultural dimension of food (7). The Food and Agriculture Organization (FAO) defines a sustainable diet as: “those diets with low environmental impacts which contribute to food and nutrition security and to healthy life for present and future generations. Sustainable diets are protective and respectful of biodiversity and ecosystems, culturally acceptable, accessible, economically fair and affordable; nutritionally adequate, safe and healthy, while optimizing natural and human resources” (8). Although this definition is widely used, the notion of sustainability is mobilized in different ways by the multiple actors involved in the agri-food system (5). This polysemy is also reflected among consumers, who often have confused or superficial perceptions of the concept and express doubts about its meaning (9–12).

Universities have a great and undeniable potential as catalyzers for sustainability, being both formal learning institutions and places where informal, mutual influences and lay/expert knowledge meet (13–15). These organizations are also fundamental to achieving the SDGs proposed by the United Nations (16–18). In fact, one of the major challenges currently facing universities is to promote and improve training to create key professionals capable of acting in accordance with the principles of sustainability (19). Understanding how university communities perceive and understand the concept of a sustainable diet is fundamental to improve training and develop policies, educational activities and individual practices aimed at sustainability awareness and application (14, 20). However, studies addressing the perceptions of sustainability in large university communities are still lacking.

Sonetti et al. (14) analyzed the representations of sustainability and the SDGs among members of a polytechnic university in Italy and reported heterogeneous and sometimes contradictory representations, as well as a less than holistic conception of sustainability, even in those who define themselves as experts on the subject (14). A study in Spain found that university students considered sustainability to be important and that sustainability training should be included in all areas and at all levels of education. However, the study participants did not know how to define the concept of sustainability, mainly associating it with recycling and the balance between production-consumption and they were unable to express a holistic view (19). Another study, conducted with teaching staff at the University of Valencia (Spain) showed that teachers had a lack of environmental knowledge and inadequate training in sustainability-related issues (13). Busquets et al. (15) also verified among professors at several Spanish universities that their perceptions of sustainability often did not cover all its dimensions and were mainly focused on environmental aspects (15).

Taking this context into account, the aim of this study was to analyze the level of knowledge and perceptions of food sustainability in a university community from Spain.

## Materials and methods

An exploratory and descriptive cross-sectional study, based on a quantitative methodology, was carried out between July and November 2021 by an interdisciplinary team composed of researchers from the Food and Nutrition Torribera Campus of the University of Barcelona (UB).

### Data production

A questionnaire was specifically designed for this study based on previous research on food, sustainability and risk perceptions (9, 12, 14). The instrument encompassed different main themes: food perception, food decisions, food concerns, food trust, level of knowledge concerning sustainability issues (environmental impact, SDGs, Green Deal, carbon footprint, biodiversity, local products, etc.), perceptions of one’s own diet pattern, perceptions of food sustainability, barriers to a sustainable diet and food waste. It consisted of 28 items, 7 of which were for the socio-economic characterization of the sample and 21 concerned the level of knowledge and perceptions related to food and sustainability. Most of the questions were Likert-type and multiple-choice with predefined response options. In addition, there were two open-ended subjective free-association questions (“Which word do you associate with the concept ‘food’?” and “Which word do you associate with the concept ‘sustainable diet’?”) (questionnaire available in Supplementary material).

The content of the questionnaire was validated using the Content Validity Index for Items (I-CVI) and Content Validity Index for Scale (S-CVI) (21–24). In accordance with these methods, nine experts from different fields related to the topic of food and sustainability (sociology, nutrition, food sciences, economics, anthropology, and public health) were invited to evaluate the questionnaire. These experts were selected from different UB research groups according to the relevance of their research in the field of food and sustainability. They evaluated each question of the questionnaire on a numerical scale from 1 to 4, considering the following aspects: relevance, simplicity, ambiguity and clarity. In addition, the experts were invited to make comments and suggestions for each question. The I-CVI for each question was obtained by adding the number of experts who gave the question a score of 3 or 4, divided by the total number of experts. The final score for each question ranged from 0 to 1, and the closer to 1, the greater the expert consensus. According to Lynn (21), for item acceptability the I-CVI should be no lower than 0.78, that is to say, every question with a lower value had to be compulsorily modified by the research team, based on the comments and suggestions of the experts. The S-CVI indicates the degree of consensus among experts regarding the relevance of the general content of the questionnaire. This index was calculated through the average of the I-CVIs for “relevance” by summing them and dividing by the number of items. The S-CVI also varies from 0 to 1, with 1 being the maximum consensus among experts regarding the relevance of the content. According to Polit and Beck (23), the criterion used for acceptability of the S-CVI was a score no lower than 0.90. Following this content validation, a pilot test was carried out online, *via* Google Forms, with 30 people from the university community. Once the questionnaire was completed, they were also able to comment and suggest changes to improve the instrument. The questionnaire was adjusted again after this pilot test to obtain its final version.

### Context and sampling

The study was conducted within the community of the UB, one of the largest universities in Spain. The UB is composed of more than 25 centers, offering 73 bachelor’s degrees and 173 university master’s degrees that cover all knowledge areas: humanities, health sciences, social sciences, experimental sciences and engineering. The UB community comprises 72,161 students, 5,963 researchers and teaching staff, and 2,387 administrative and service employees.

For the study, all individuals working or studying in this university were invited to answer the questionnaire (convenience sample). No exclusion criteria were established with respect to the participant gender, age, faculty/center/scientific background, place of residence, and nationality. For the characterization of the sample, data were collected on gender, age, educational level, occupation (student, professor, or administrative staff), faculty or center affiliation, and average monthly household income.

The questionnaire was administered online between October and November 2021 *via* Google Forms and sent by email to all members of the UB community with the support of the university’s administrative services. A total of 1,225 responses were obtained, 5 of which were excluded after a sensing cleaning data procedure checking for duplicates and missing data, resulting in a final data set of 1,220 responses.

### Data analysis

Textual data collected with the free association questions were first pre-processed to reduce data dispersion; synonyms and multi-words were identified, and verbs were reduced to the infinitive. Four experts from the research team classified the 178 words mentioned by participants into nine analytical categories according to their semantic field. Chi-squared tests were performed for categorical variables to examine whether the proportion of participants was different across gender or affiliations (administrative staff, teaching staff, and students). When mean scores were analyzed, two-tailed independent sample *t*-tests were carried out to explore the differences between male and female and analyses of variance (ANOVA) when exploring differences among affiliations. *Post-hoc* tests were performed when differences among affiliations were detected. Spearman correlations were carried out to test the putative relationship between perceptions of sustainability and healthiness. All these analyses were performed using SPSS for Windows 24.0. For the statistical analyses according to gender, considering the low rate of participants who indicated “others” in the questionnaire, only the participants who defined themselves as men and women were considered. In this sense, we refer to “sex” in the results.

### Ethical aspects

The study was conducted according to the recommendations of the Code of Good Practice in Research of the University of Barcelona (25) and in accordance with the ethical standards laid out in the 1964 Declaration of Helsinki and subsequent updates (26). The questionnaire was anonymous, and participants accessed information about the study on the first page of the online survey, expressing their consent to participate by ticking “accepted.”

## Results

### Participant profile

Out of the 1,220 participants (accounting for around a 2% of this university population) who completed the questionnaire, 68.3% were female, and most of them were between 51 and 65 years old (46.8%), teaching staff (48.3%), and from the health sciences academic field (33.2%). On the contrary, although students represent the largest group at the UB, they were underrepresented among the participants of this study (17.8%), which may reflect a low level of interest of this subgroup in the research topic and/or low engagement with this kind of initiatives promoted by the university. Moreover, most participants were from the field of health sciences, which may be related to their greatest awareness to health-related topics and to the affiliation of most researchers of the study. Table 1 presents the effective response rates (i.e., percentage of potentially eligible affiliates who participated) distribution of the sample and its characteristics according to their affiliation (administrative staff, teaching staff or students).

**TABLE 1:** Socio-demographic characteristics of the analyzed university community.

	Total		Affiliation (%)		
	*n*	%	Adm. Staff (*n* = 414)	Teach. Staff (*n* = 589)	Students (*n* = 217)
**Sex**					
Male	380	31.7	29.2	59.7	11.1
Female	819	68.3	36.6	42.6	20.8
**Age range (years)**					
18–30	233	19.1	7.3	5.2	87.6
31–50	382	31.3	38.5	58.4	3.1
51–65	571	46.8	48.3	56.0	0.2
>66	34	2.8	0.0	100.0	0.0
Academic field*					
Arts and humanities	148	12.1	34.5	62.2	3.4
Sciences	224	18.4	28.1	69.6	2.2
Health sciences	405	33.2	16.5	34.1	49.4
Social sciences	254	20.8	21.7	77.6	0.8
Technical services and associated centers	189	15.5	33.9	48.3	17.8

*Classification according to The National Agency for Quality Assessment and Accreditation of Spain, ANECA.

### Level of knowledge of sustainability

In general, most participants (70.4%) indicated that they had often heard about the environmental impact of food (only 4.8% had not). No statistical significant differences were found by sex (70.0% male and 70.2% female). In contrast, the analysis by affiliation showed statistical significant differences: the teaching staff were more likely to state that they had often heard about the environmental impact (76.6%) in comparison with administrative staff (71.3%) and especially in comparison with students (52.1%) (χ^2^ = 73.71 and *p* < 0.001). When participants were asked if they knew about the SDGs of the UN, a statistical difference was observed related to sex more male (70.8%) than female participants (64.1%) indicated awareness of this concept (χ^2^ = 5.18 and *p* = 0.023). Important differences were also identified among affiliations: teaching staff (78.1%) showed a higher level of knowledge than administrative staff (65.2%) and students (34.6%) (χ^2^ = 134.08 and *p* < 0.001). Regarding the Green Deal, it is worth noting that 56.8% of the sample did not know about this European strategy; moreover, following the same pattern as in the previous questions, teaching staff (49.6%) presented a significantly higher level of knowledge than the other two groups (37.0% administrative staff and 37.8% students) (χ^2^ = 18.93 and *p* < 0.001).

**FIGURE 1:**
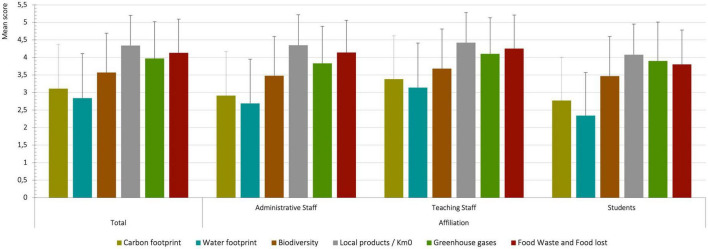
Level of knowledge of concepts related to sustainability.

When informants were asked to evaluate from 1 to 5 their level of knowledge regarding specific concepts (“carbon footprint,” “biodiversity,” “greenhouse gases,” etc.), the results indicated that participants tended to be more familiar with more general and less technical concepts, such as “local products/Km0” (common expression in Spanish-speaking populations that referred to local foods that have not traveled far after production) ($\bar{X}$ = 4.34, *SD* = 0.86) and “food waste/food lost” ($\bar{X}$ = 4.13, *SD* = 0.96). No significant differences were found by sex. Regarding the affiliations, once again teaching staff declared a higher level of knowledge than the other two groups, especially in comparison with students (Figure 1).

### Food decisions and social perceptions of food sustainability

Almost all participants declared that their diet is often healthy (99.7%) and sustainable (96.3%). No statistical significant differences were observed according to sex or affiliation. Furthermore, 96.1% of the participants stated that a healthy diet corresponds to a sustainable diet.

Taking all the participants together, the factors that most influenced their eating decisions were the quality, ingredients, and nutritional composition of food (38.0%), followed by pleasure and taste (35.9%), and preventing chronic illness and its effect on health (33.0%). Factors related to food sustainability were not prioritized when food decisions were made: the origin of food and support for the agro-ecological territory (13.4%), ecology, environment, or animal welfare (8.5%) and seasonality (seasonal products) (5.2%). No statistical difference was found according to sex. The analysis by affiliation indicated several statistical significant differences, revealing that the rationalities mobilized in food choice vary according to the professional/occupational status: price was more important for the administrative staff (12.1%) than for students (10.1%) and teaching staff (7.3%) (χ^2^ = 6.65 and *p* = 0.036); concerns about body weight or physical shape had more influence on students (10.1%) than administrative staff (5.3%) and teachers (3.2%) (χ^2^ = 15.49 and *p* < 0.001); the origin of food and support for the agro-ecological territory was taken into consideration far more by administrative staff (16.2%) and teaching staff (14.6%) than by students (5.1%) (χ^2^ = 16.42 and *p* < 0.001); the seasonality (seasonal products) was more relevant for teaching staff (7.1%) and administrative staff (4.3%) than for students (1.8%) (χ^2^ = 9.93 and *p* = 0.007); pleasure and taste was more important for students (48.8%) than teaching staff (37.2%) and administrative staff (27.3%) (χ^2^ = 29.55 and *p* < 0.001); preventing chronic illness and the effect on health had more impact on teaching staff (37.7%) and administrative staff (33.3%) than students (19.4%) (χ^2^ = 24.18 and *p* < 0.001); state of mind played a bigger role for students (9.2%) than teaching staff (2.7%) and administrative staff (4.1%) (χ^2^ = 16.20 and *p* < 0.001).

In general, when participants were specifically asked to indicate on a scale from 1 to 5 the importance given to food sustainability at the time of purchasing foodstuffs, the answers also showed that it was not a key motivation: the total average among all participants was 2.42 (*SD* = 0.82) and the average for either gender or affiliation, considered separately, reached 3 points. Statistical significant differences were found between the sexes and among affiliations. Male participants gave higher values ($\bar{X}$ = 2.48, *SD* = 0.86) than females ($\bar{X}$ = 2.38, *SD* = 0.79) (*F* = 6.06 and *p* = 0.014) and, contrary to the question in the previous paragraph, students gave higher values ($\bar{X}$ = 2.75, *SD* = 0.90) than administrative staff ($\bar{X}$ = 2.43, *SD* = 0.77) and teaching staff ($\bar{X}$ = 2.29, *SD* = 0.79) (*F* = 26.0 and *p* < 0.001).

The food-related factors that concern the university community were also analyzed (Table 2). The three aspects that concerned the sample the most were: food waste/food lost ($\bar{X}$ = 4.15, *SD* = 0.95), hygienic conditions at home and outside the home ($\bar{X}$ = 4.06, *SD* = 0.99) and use of plastic and plastic packaging ($\bar{X}$ = 4.01, *SD* = 0.95). The three aspects that generated the least concern in the university community were: genetically modified organisms (GMOs) ($\bar{X}$ = 3.12, *SD* = 1.37), allergens ($\bar{X}$ = 2.77, *SD* = 1.42) and the presence of gluten and/or lactose ($\bar{X}$ = 2.07, *SD* = 1.34). Although the three aspects that most or least concerned female and male participants were almost the same, female participants indicated higher levels of concern for 17 of the 18 items proposed (the exception was weight gain) and statistical significant differences were observed in 15 of the 18 items. Among the affiliations, administrative staff showed greater concern for every factor except the presence of contamination by viruses (avian flu, norovirus, SARS-CoV-2, etc.) and bacteria (salmonella, listeria, etc.). Statistically significant differences were observed between affiliations for all the factors included in the survey (*p* < 0.05).

**TABLE 2:** Level of concern for different food-related factors.

	Total	Sex		*P*-value	Affiliation			*P*-value
	Mean (SD)	Male	Female		Adm. Staff	Teach Staff	Students	
Pesticides	3.77 (1.12)	3.63 (1.27)	3.83 (1.18)	< 0.001	4.01 (1.10)	3.83 (1.19)	3.12 (1.27)	< 0.001*
Hygiene in the home and outside the home	4.06 (0.99)	3.91 (0.98)	4.14 (0.99)	< 0.001	4.16 (0.94)	3.96 (1.03)	4.15 (0.95)	< 0.001^ab^
Contamination by viruses and bacteria	3.78 (1.20)	3.55 (1.22)	3.90 (1.17)	< 0.001	3.96 (1.14)	3.58 (1.23)	3.99 (1.11)	< 0.001^ab^
Allergens	2.77 (1.42)	2.53 (1.34)	2.89 (1.44)	< 0.001	3.08 (1.40)	2.66 (1.38)	2.46 (1.44)	< 0.001^ac^
Presence of gluten and/or lactose	2.07 (1.34)	1.74 (1.09)	2.23 (1.41)	< 0.001	2.40 (1.39)	1.92 (1.26)	1.85 (1.31)	< 0.001^ac^
Residues of antibiotics and hormones in animal products	3.58 (1.28)	3.25 (1.31)	3.74 (1.23)	< 0.001	3.85 (1.20)	3.57 (1.26)	3.12 (1.33)	< 0.001*
Presence of chemical contaminants	4.00 (1.17)	3.87 (1.21)	4.07 (1.14)	< 0.001	4.17 (1.11)	3.99 (1.14)	3.73 (1.26)	< 0.001*
Animal welfare	3.73 (1.12)	3.50 (1.18)	3.83 (1.06)	< 0.001	3.95 (1.05)	3.61 (1.10)	3.64 (1.22)	< 0.001^ac^
Genetically modified organisms	3.12 (1.37)	2.87 (1.40)	3.24 (1.34)	< 0.001	3.59 (1.28)	3.03 (1.36)	2.45 (1.23)	< 0.001*
Chronic non-communicable diseases	3.77 (1.19)	3.66 (1.20)	3.83 (1.18)	0.020	3.97 (1.12)	3.68 (1.18)	3.67 (1.28)	< 0.001^ac^
Weight gain	3.70 (1.12)	3.70 (1.03)	3.70 (1.12)	0.980	3.89 (1.02)	3.65 (1.08)	3.45 (1.33)	< 0.001^ac^
Sugar and salt content	3.83 (1.03)	3.77 (1.06)	3.87 (1.00)	0.120	3.95 (0.96)	3.84 (1.01)	3.59 (1.14)	< 0.001^bc^
Fat and saturated fat content	3.94 (0.97)	3.82 (1.02)	4.00 (0.94)	< 0.001	4.06 (0.91)	3.99 (0.94)	3.60 (1.10)	< 0.001^bc^
Food additives	3.42 (1.19)	3.22 (1.20)	3.52 (1.17)	< 0.001	3.69 (1.06)	3.47 (1.17)	2.79 (1.26)	< 0.001*
Food waste	4.15 (0.95)	4.00 (1.02)	4.22 (0.90)	< 0.001	4.29 (0.85)	3.80 (1.14)	4.15 (0.95)	< 0.001^bc^
Use of plastic and plastic packaging	4.01 (1.03)	3.84 (1.11)	4.09 (0.98)	< 0.001	4.12 (0.97)	4.05 (0.96)	3.68 (1.24)	< 0.001^bc^
Environmental impact	3.73 (1.14)	3.58 (1.17)	3.79 (1.11)	< 0.001	3.87 (1.09)	3.72 (1.09)	3.47 (1.29)	< 0.001^bc^
Socioeconomic situation of local agriculture	3.73 (1.12)	3.67 (1.12)	3.77 (1.11)	0.130	3.93 (1.04)	3.80 (1.06)	3.15 (1.27)	< 0.001^bc^

*Indicates statistically significant differences among the three different groups of affiliation.

^a^Indicates statistically significant differences between administrative staff and teaching staff.

^b^Indicates statistically significant differences between teaching staff and students.

^c^Indicates statistically significant differences between administrative staff and students.

In this article the results of two main questions exploring the social perceptions of the concept “sustainable diet” were analyzed (an open-ended question “Which word do you associate with the concept ‘sustainable diet’?” and a multiple choice question “Which are the three most important aspects for a sustainable diet?”). One hundred seventy-eight different words were associated with “sustainable diet,” among which the five most frequently cited were: proximity (mentioned 145 times), ecological (136), environment (115), balance (63) and future (58). The categorization of these words revealed that participants associated a sustainable diet with words most related to values (responsible, kindly, etc.) and to the environmental dimension of sustainability such as: planet, environment, ecology, nature, etc. (Table 3). A third large category of words involved the idea of proximity (e.g., locality, Km0, etc.). It is worth mentioning that for this item, participants did not focus on social and economic dimensions in their answers.

**TABLE 3:** Frequency (%) of word categories associated with the concept of “sustainable diet” through the free association task.

Category name	N*	%
Values (e.g., adequate, responsible, kindly, conscious, intelligent)	280	23.0
Environment (e.g., nature, environment, planet, earth)	253	20.7
Proximity (e.g., local, Km0, seasonal)	203	16.6
Sustainable production (e.g., ecologic, organic, free from contaminants, artisanal, agroecology, circular)	122	10.0
Future (e.g., future, durable, conservation, preservation)	94	7.7
Health (e.g., healthy, nutrition, plant-based, vegan, wellbeing)	89	7.3
SDGs (e.g., food waste, climate change, reuse, sustainability)	70	5.7
Socioeconomics (e.g., justice, economy, equity)	55	4.5
Difficulties/criticism (e.g., price, expensive, impossible, fashion, utopia, laziness, inaccessible)	45	3.7

*Nine participants (0.8%) did not answer properly or indicated that they did not know.

When participants were asked to choose the most important aspects involved in following a sustainable diet (Table 4), the three most chosen options were the presence of locally produced, seasonal products (71.8%), that the diet was respectful of ecosystem biodiversity and had a low environmental impact (68.6%), and no or minimum food waste (37.7%). No significant differences were found between males and females. Regarding the affiliations, statistically significant differences were found for 6 out of the 10 aspects (*p* < 0.05). In general, administrative and teaching staff gave similar responses and were differentiated from the students, who gave more importance to locally produced food (81.6%), biodegradable and compostable packaging (46.1%), and the monetary cost (28.1%).

**TABLE 4:** Distribution (%) of answers about the most important aspects of a sustainable diet.

	Total		Sex (%)		*P*-value	Affiliation (%)			*P*-value
	*n*	%	Male	Female		Adm. Staff	Teach. Staff	Students	
Rich in plant-based foods	106	8.8	10.8	7.9	0.105	8.2	12.4	1.8	<0.001
With no or the minimum amount of food waste	450	37.7	39.2	36.8	0.413	38.4	37.9	33.6	0.463
With biodegradable, compostable packaging	372	31.0	28.2	32.4	0.144	31.9	25.6	46.1	<0.001
With locally produced, seasonal products	863	71.8	74.5	70.8	0.190	67.1	71.5	81.6	0.001
Respectful of ecosystem biodiversity and with a low environmental impact	822	68.6	69.2	68.3	0.740	69.8	69.1	65.0	0.434
With products from companies that respect workers’ social rights	165	13.8	25.5	15.0	0.064	14.7	13.8	12.9	0.807
Affordable	250	20.9	21.8	20.4	0.565	20.0	18.8	28.1	0.014
Organic/ecological	186	15.5	12.6	16.8	0.060	16.7	16.6	9.2	0.022
Simple, without additives, based on foods with few ingredients and little processed	339	28.3	30.0	27.5	0.366	28.0	31.7	18.0	0.001
Culturally acceptable	43	3.6	2.6	4.0	0.226	4.8	2.5	3.7	0.153

**FIGURE 2:**
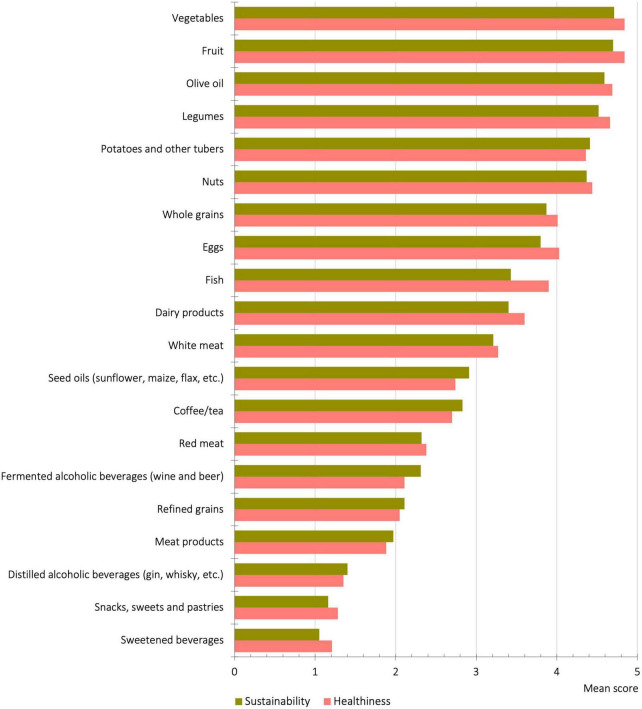
Perceptions regarding the level of sustainability and healthiness of different foods.

Participants were also asked to rate from 1 to 5 different foodstuffs regarding their healthiness and sustainability value (Figure 2). It should be mentioned that no correlation was found for these two aspects with sex or affiliation. In general, the three products perceived as most sustainable were vegetables ($\bar{X}$ = 4.71, *SD* = 0.75), fruit ($\bar{X}$ = 4.70, *SD* = 0.76), and olive oil ($\bar{X}$ = 4.59, *SD* = 0.84). The products perceived as the least sustainable were distilled alcoholic beverages ($\bar{X}$ = 1.40, *SD* = 1.22), snacks, sweets, and pastries ($\bar{X}$ = 1.16, *SD* = 1.06) and sweetened beverages ($\bar{X}$ = 1.05, *SD* = 1.03). Between the genders, statistically significant differences in the perceived sustainability value were found in the case of olive oil (male $\bar{X}$ = 4.53, female $\bar{X}$ = 4.64, *t* = 2.16 and *p* = 0.031), potatoes (male $\bar{X}$ = 4.30, female $\bar{X}$ = 4.45, *t* = 2.55 and *p* = 0.011), nuts (male $\bar{X}$ = 4.28, female $\bar{X}$ = 4.42, *t* = 2.22 and *p* = 0.027), whole grains (male $\bar{X}$ = 3.76, female $\bar{X}$ = 3.93, *t* = 2.10 and *p* = 0.036), fermented alcoholic beverages (male $\bar{X}$ = 2.45, female $\bar{X}$ = 2.25, *t* = 2.42 and *p* = 0.016), and refined grains (male $\bar{X}$ = 2.27, female $\bar{X}$ = 2.04, *t* = 2.67 and *p* = 0.008). Among affiliations, statistically significant differences were found in red meat (*F* = 4.30 and *p* = 0.014), fish (*F* = 5.31 and *p* = 0.005), dairy products (*F* = 3.78 and *p* = 0.023), eggs (*F* = 11.17 and *p* < 0.001), nuts (*F* = 8.09 and *p* < 0.001), refined grains (*F* = 5.11 and *p* = 0.006), legumes (*F* = 5.19 and *p* = 0.006), olive oil (*F* = 8.75 and *p* < 0.001), snacks (*F* = 3.46 and *p* = 0.032), coffee and tea (*F* = 3.09 and *p* = 0.046), and fermented alcoholic beverages (*F* = 16.69 and *p* < 0.001).

Participants were also asked to rate from 1 to 5 the extent to which different factors could hinder them in following a sustainable diet (Table 5). The total average scores for all the factors were higher than 3, which may indicate that all of them can impede the implementation of sustainable practices. For the whole sample, in order of importance, the three factors considered to be the main barriers were: cost ($\bar{X}$ = 4.26, *SD* = 0.87), lack of information ($\bar{X}$ = 4.14, *SD* = 0.95), and ease of purchase (accessibility) ($\bar{X}$ = 3.99, *SD* = 0.98). No significant differences were observed between male and female informants and among the three affiliations.

**TABLE 5:** Factors perceived as barriers to following a sustainable diet.

	Total	Sex		*P*-value	Affiliation			*P*-value
	Mean (SD)	Male	Female		Adm. Staff	Teach. Staff	Students	
Cost	4.26 (0.87)	4.14 (0.94)	4.31 (0.839	0.319	4.33 (0.85)	4.21 (0.86)	4.25 (0.92)	0.112
Lack of information	4.14 (0.95)	3.97 (1.00)	4.22 (0.91)	0.160	4.16 (0.94)	4.11 (0.95)	4.15 (0.97)	0.738
Lack of culinary knowledge	3.35 (1.19)	3.31 (1.15)	3.38 (1.21)	0.059	3.46 (1.19)	3.29 (1.19)	3.31 (1.18)	0.680
Lack of time	3.62 (1.21)	3.57 (1.23)	3.64 (1.21)	0.436	3.68 (1.23)	3.61 (1.20)	3.52 (1.21)	0.291
Food preferences and taste	3.23 (1.21)	3.34 (1.18)	3.18 (1.22)	0.545	3.24 (1.18)	3.21 (1.23)	3.29 (1.23)	0.670
Food traditions	3.45 (1.15)	3.45 (1.14)	3.45 (1.15)	0.928	3.47 (1.14)	3.44 (1.14)	3.42 (1.19)	0.859
Accessibility to food	3.98 (0.98)	3.86 (0.95)	4.04 (0.98)	0.640	3.97 (1.00)	3.95 (0.96)	4.07 (0.99)	0.316

### Food waste

Two questions specifically addressed the issue of food waste. The first one focused on the frequency with which participants waste food, in which they were asked to evaluate their food waste from 1 to 4. Among all the participants, the mean frequency score was 2.56 (*SD* = 0.70) out of 4, without significant differences according to sex or affiliation. The second question concerned the actions carried out by participants to avoid food waste (Table 6). Planning shopping and meals ($\bar{X}$ = 4.72, *SD* = 0.58), reusing leftovers ($\bar{X}$ = 4.60, *SD* = 0.70) and making a shopping list ($\bar{X}$ = 4.37, *SD* = 0.94) are the three actions most employed by the informants. Four statistical significant differences were found between male and female individuals: reusing leftovers (*t* = 5.82 and *p* < 0.001), planning shopping and meals (*t* = 3.63 and *p* < 0.001), making a shopping list (*t* = 5.17 and *p* < 0.001) and consuming foods that last longer (frozen foods, preserves) (*t* = 0.90 and *p* = 0.008). Significant differences were observed among the three affiliations in six of the nine options proposed in the questionnaire (*p* < 0.05): planning shopping and meals, making a shopping list, learning cooking techniques to preserve foods, making organic compost, consuming foods that last longer (frozen foods and preserves) and taking part in initiatives to recover food.

**TABLE 6:** Actions to avoid food waste at home.

	Total	Sex (%)		*P*-value	Affiliation (%)			*P*-value
	Mean (SD)	Male	Female		Adm. Staff	Teach. Staff	Students	
Use leftovers	4.60 (0.67)	4.44 (0.75)	4.68 (0.61)	< 0.001	4.60 (0.69)	4.63 (0.64)	4.53 (0.71)	0.163
Plan shopping and meals	4.72 (0.58)	4.63 (0.65)	4.76 (0.54)	< 0.001	4.78 (0.46)	4.72 (0.57)	4.59 (0.75)	0.001^bc^
Write shopping lists	4.37 (0.94)	4.17 (1.02)	4.46 (0.88)	< 0.001	4.51 (0.80)	4.30 (0.99)	4.29 (0.98)	0.001^ac^
Buy smaller quantities of food	3.98 (1.04)	3.85 (1.05)	4.05 (1.02)	0.480	4.08 (1.02)	3.98 (1.02)	3.80 (1.12)	0.007^c^
Learn cooking techniques to preserve foods	3.92 (1.04)	3.78 (1.04)	3.99 (1.02)	0.266	4.10 (0.97)	3.77 (1.07)	3.98 (1.00)	< 0.001^ab^
Make organic compost	2.96 (1.24)	2.76 (1.20)	3.05 (1.24)	0.950	3.04 (1.26)	2.79 (1.21)	3.24 (1.21)	< 0.001^ab^
Consume foods that last longer	2.90 (1.16)	2.95 (1.10)	2.89 (1.19)	0.008	2.94 (1.24)	2.74 (1.09)	3.27 (1.14)	< 0.001*
Take leftovers home from a restaurant	3.54 (1.21)	3.36 (1.18)	3.63 (1.21)	0.266	3.58 (1.19)	3.46 (1.20)	3.65 (1.25)	0.087
Take part in initiatives to recover food	3.31 (1.25)	3.04 (1.26)	3.44 (1.23)	0.657	3.36 (1.23)	3.11 (1.25)	3.73 (1.20)	< 0.001*

*Indicates statistically significant differences among the three affiliations.

^a^Indicates statistically significant differences between administrative staff and teaching staff.

^b^Indicates statistically significant differences between teaching staff and students.

^c^Indicates statistically significant differences between administrative staff and students.

## Discussion

To our knowledge, this is the first study to analyze the level of knowledge and social perceptions of food sustainability of a whole university community in Spain. The study was carried out with the teaching staff, administrative staff and students of the UB, one of the largest universities in the country. Overall, the results indicated that a greater effort is needed to enhance knowledge of food sustainability and to increase the importance given to this dimension when food choices are made.

### Level of knowledge of sustainability

Although most participants declared that they had often heard about the environmental impact of food or the SDGs, the level of knowledge was lower for specific or more technical aspects of sustainability, such as: “carbon footprint,” “biodiversity,” or “greenhouse gases.” Respondents had higher levels of knowledge of concepts that appear more frequently in the media and that can be directly applied in personal practices, such as local food/Km0 and food waste. These concepts feature in many of the recommendations made by different institutions for the implementation of sustainable individual practices in Catalonia, such as the report Sustainable food: a handbook for cities by the Barcelona City Council (27). Burkhart et al. (28) also observed that Australian dietetic students were familiar with and concerned about sustainability, but in a superficial way, since the level of familiarity was low when specific factors encompassed by the concept were assessed. García-González et al. (9) identified a similar situation in a Spanish sample: the most recognized concepts were “environmental impact” and “local food,” whereas the least familiar were “carbon footprint” and “green water/blue water.” Moreover, in the present study, the level of knowledge of topics related to sustainability depended mainly on the affiliation: while teaching staff presented a higher level of knowledge for almost all the aspects included in the questionnaire, students showed the lowest levels. Sonetti et al. (14) also found that researchers and professors from an Italian polytechnic university had the highest levels of knowledge of the SDGs, followed by postdoc and PhD students, the technical and administrative staff and students. This difference may be related to the level of formal education, as previous studies have revealed that the higher the level of education, the greater the understanding of sustainability (29, 30). Furthermore, this difference may also be related with the age of the participants, given that in a previous study with a general Spanish population, younger individuals were less motivated to adapt their diet to achieve a more sustainable pattern (29). Indeed, it is notable that students represent the largest group at the UB, but they had the lowest response rate to the survey. These data suggest that it would be worthwhile to develop strategies focused on improving the knowledge and motivation of students.

### Food decisions and social perceptions of food sustainability

Almost all the sample perceived their food-associated practices to be sustainable. This self-perception of diet can be a barrier to motivation to make changes toward a more sustainable diet. Indeed, the results regarding food decisions indicated that factors related to sustainability were not prioritized and that overall this issue was not taken into account when purchasing food. The three most important factors that influenced participant food choices were: the quality, ingredients, and nutritional composition, followed by pleasure and taste and preventing chronic illness and the effect on health. According to the Eurobarometer on perceptions of food sustainability, when making their food purchases, Europeans prioritize taste, food safety and cost over sustainability concerns (31). According to Díaz-Méndez (32), the priorities of people who cook in Spanish households are: having a varied, balanced and tasty diet, and eating in company (32). Her study found that health and taste predominate in Spanish food decisions. The present study did not reveal statistical significant differences according to sex in factors that influence food choice, but important differences were observed among the affiliations. Students, who were the youngest group, expressed concerns about the body and the hedonic and emotional dimensions of food choice to a greater extent than the other two affiliations. The latter were more influenced by health-associated issues and took sustainability-related factors more into account. These differences may be associated with age and level of education. The Eurobarometer indicated that the younger the respondent, the less likely they are to cite food safety as important compared to those who are aged 55 and over. Furthermore, among Europeans, the longer the respondent remained in education, the more likely they were to state that nutrient content is important in food choice (31).

The analysis of food-related issues of most concern for the university community revealed that in this case, sustainability-related factors, mainly related to food production, generated more concern than nutritional aspects. According to Contreras (33), the main food-associated problem facing Western industrialized society a century ago was scarcity, but the increased productivity following the Green Revolution and food industrialization has led to the emergence of a new set of issues related to food excesses, globalized food crises or food waste, engendering new concerns about production methods and their impact on the environment. Beck (34) states that with the industrialization of society, risks have become a constant in the daily lives of individuals in Western countries (34), especially concerning food consumption (35, 36). In lay social perceptions, foods derived from the industrial agri-food system are often associated with toxicity and are viewed as harmful to human and planetary health (37–39). Adamiec (40) further notes that beyond contemporary morals that encourage individuals to feel responsible for their health and their bodies, another morality is being forged that makes them feel responsible for their social and natural environment. Consequently, new food concerns and discourses have emerged (40).

The Eurobarometer on the perceptions of food risks confirms that Europeans associate them above all with chemicals and pesticides applied in production, antibiotics used in breeding, pollutants such as mercury and dioxins, animal welfare, etc., to the detriment of nutritional concerns (41, 42). In general, the concerns and perceptions related to food risks in Catalonia seem to follow the European trend (43). According to the Barometer of Food Safety in Catalonia (44), in 2015, compared to previous years, there was a higher perceived frequency of food risks, especially in relation to fruit or vegetables carrying pesticide residues (45). Another report indicated that food safety is no longer associated so much with problems related to access to food or its nutritional composition (such as fat content), but rather with the health and hygiene dimension and with the contamination and toxicity of products from production practices (46). This study revealed that a significant proportion of informants were suspicious and critical of the intensive agricultural production model, the use of pesticides and the techniques used in modern farming, such as the use of hormones. The insecurity derived from environmental pollution and production methods thus represented major concerns for the Catalan population.

However, it should be noted that food perceptions or food concerns are not always reflected in the day-to-day behaviors of individuals (47, 48), which are conditioned by a multitude of social, cultural, economic, symbolic and material aspects (33, 49). In fact, it is notable that the issue that generated most concern among the participants of the present study was wasting food, a widespread practice in the general population. According to the Ministry of Agriculture, Fisheries and Food Panel for the quantification of food waste in households, in 2020, three out of four Spanish households wasted some food and wastage reached 1,363 million kg or liters of food and beverages (50).

Female participants expressed higher levels of concern for 17 out of the 18 options proposed (the exception was weight gain). This stronger concern among women may be associated with differential historically and socially constructed gender roles (51). Women are more likely to establish a relationship between food and health, be involved in reproductive care and feeding activities at home (especially in relation to child nutrition education), internalize more food and nutrition recommendations, as well as control their diet (42, 51–54). The fact that almost 70% of the sample is made up of women may itself be an indication that this is a subject (food, health, or sustainability) that arouses greater interest and concern in the female than the male collective. However, it is surprising that no gender difference was found for concerns related to weight gain, considering that most scientific literature shows that the female body is more subjected to normativization and aesthetic pressure (55, 56).

Analyzing the perceptions of what constitutes a sustainable diet revealed that the social representations of the participants are not very holistic and are mainly associated with the ecological dimension at the cost of social and economic dimensions. The perceptions of the university population analyzed in this study are in this sense similar to those of the Spanish population in general. García-González et al. (9) point out that although the FAO definition of a sustainable diet emphasizes that sustainable food should not only be environmentally friendly, but also culturally acceptable, accessible, and economically fair, these aspects are underestimated by the Spanish population (9). Research with university samples in Spain and other countries has also found that this population lacks a holistic view of the concept of a sustainable diet (13–15, 19). These results suggest that it is crucial to develop training activities to foster a broader and more complex conception of food sustainability among the university community. Moreover, the promotion of knowledge and sustainable practices may operate not only through teaching activities and research but also through actions that seek social impact and transformation, as well as the co-management of the university environment itself (57, 58).

Participants associate a sustainable diet above all with the consumption of local and seasonal products, respect for ecosystem biodiversity, a low environmental impact, and no or a minimum amount of food waste. It is worth noting that local products and food waste were also the topics that the participants knew most about, and that receive widespread attention in the media. The theme of local products in particular seemed to be of great importance to the participants. In studies with participants from different cultural backgrounds, including in Spain, local or proximity products have been increasingly valued and associated with trust, good quality and health (36, 39). The interest in local products is a reaction to the transformations provoked by the industrial food system that cause a weakening of the links between food and territory and among the eater and food and the natural/cultural environment (37, 59). “Eating local” would allow a return to tradition and the know-how that individuals are afraid of losing in a globalized society (60) and it would be a way to rediscover a sense of security with respect to modern food (59). Moreover, studies conducted in Spain have observed an increase in the purchase of food locally produced and/or sold through short-circuit retail during the COVID-19 pandemic (61–63). The Barometer of the Government of Catalonia and the Promoter of Catalan Exports (Prodeca) has reflected this trend by revealing that 37% of Catalan individuals bought more local products during the pandemic (only 8% bought less) (64). This phenomenon may be related to changes in perceptions of the agri-food chain that occurred during this period, and which has led to the questioning of the global agri-food production and distribution system and greater solidarity with and appreciation of local producers (63, 65).

With regard to the perception of different foods in terms of their healthiness and sustainability, in both cases, there is a more positive assessment of foods of plant origin, especially fruit, vegetables and olive oil, in concordance with their lower environmental impact (66), at the cost of foods of animal origin, mainly red meat and processed products. These results corroborate the analyses of other studies carried out in different socio-cultural contexts (9, 12, 36, 39). Fruit and vegetables are perceived as healthier and more sustainable because of their nutritional composition (rich in vitamins, minerals and fiber), because they are considered fresh and natural, and they are products that generate a link with regional or national agriculture (40, 67, 68). In general, studies also reveal a positive perception of olive oil (59, 69). Likewise, the Spanish population seems to associate itself directly with the Mediterranean Diet, which in their social representations evokes healthiness, tradition, proximity, and sustainability (70).

The consumption of meat and dairy products has been questioned due to the impact of their production on the environment (1, 71) and this problematization seems to be reflected in the perceptions of the studied university community. In all historical periods and socio-cultural groups, animal products, mainly red meat, are shrouded in ambiguous and ambivalent discourses (59, 64). Fischler (37) notes that meat is at the same time the most sought-after food for humans, and the most abhorred food. Moreover, meat is attached to contradictory meanings within Western societies (52). This ambivalence seems to be linked to objective aspects, associated with the nutritional composition and the effect on health, but also to subjective, symbolic and ethical aspects concerning animal welfare, the environment and man’s relationship with animals and death. Moreover, this perception of meat has intensified in recent years following the World Health Organisation [WHO] (72) on the consumption of meat products and the prevalence of diseases (72), but also as a consequence of the discourses that warn about the impacts on the environment (1, 3).

As a whole, the data on food perceptions are indicative that the studied population has internalized institutional discourses regarding both nutrition and sustainability. Recommending a lower consumption of animal and processed foods while promoting plant-based diets is widespread, including in public health recommendations in Spain (73) as well as in the Eat-Lancet Report (3) or even the IPCC (74). However, data on individual practices confirm that internalized knowledge and norms are not always reflected in day-to-day practices, which are complex and conditioned by a multitude of factors, as observed in this study. It is noteworthy that the percentage of the population following a vegetarian or vegan diet has increased in the last decade (75) as social awareness of the environmental impact of food has also grown. However, meat consumption must be reduced still further to align human health with planetary health (66). A study carried out in Barcelona, for example, has shown that CO2 equivalent emissions generated by food and drink consumption amount to 2.5 million tons per year. Domestic food consumption by residents is responsible for 3/4 of the emissions. Some foods, such as meat, dairy, eggs and seafood, are targeted as the most problematic, being responsible for about 60% of the carbon footprint of household consumption. According to the authors of the study, if 25% of the city’s residents cut back their consumption of animal protein, emissions would be reduced by 285,000 tons of CO_2_ equivalents, corresponding to an 11% decrease in the city’s carbon footprint (76).

All the factors proposed as possible barriers to sustainable diets received scores of more than three out of five, revealing that following a sustainable diet is perceived as difficult. Corroborating previous studies, these aspects include price, lack of information and accessibility. A lack of money is seen as central to not being able to eat well in general, but especially healthy and sustainable food (33). Likewise, the price of food considered sustainable (organic, local, etc.) represents a barrier to sustainable eating, even for those who are not in a situation of poverty. Studies indicate that price is a crucial factor in the decision to buy sustainable products (11, 77, 78). According to a recent report on perceptions of organic products issued by the Catalan Government, among the factors that would encourage the purchase of organic products, a more affordable price clearly stands out (57.7%). The survey also reveals that among citizens who are familiar with organic products but do not consume them, price is the most cited reason (53.4%). Concerning the level of consumer information, research conducted in different Spanish cities shows that several factors limit the transition process from internalized values and knowledge to their application in sustainable purchasing decisions and practices, among which the level of education and information stand out. According to Eldesouky et al. (79), higher levels of consumer education tend to go hand in hand with a better understanding of environmental issues, and these consumers even show a higher degree of sensitivity or willingness to consider them as relevant attributes in their purchases (79). The accessibility of sustainable products is another important factor. The Eurobarometer on perceptions of food sustainability reveals that almost half of the respondents (especially individuals from disadvantaged social classes) state that affordability of healthy and sustainable food (49%) would help them to adopt a healthy and sustainable diet and for 45% having healthy and sustainable food options available where they usually buy food would help them adopt a healthy and sustainable diet (31). Accessibility can be directly related to the social environment and where people live. A study conducted in Barcelona’s metropolitan area on the characteristics of the city’s food environments and access to shops with organic products found that access to these shops is unevenly distributed across the city and is conditioned by the socio-economic status of neighborhoods (80).

### Food waste

The final part of the questionnaire dealt with the issue of food waste. The mean score for the frequency with which food is wasted among all the participants was 2.56 out of 4 (without differences according to sex or affiliation), indicating that wasting food is a widespread practice, although most participants expressed concern about it and considered its reduction to be very important for food sustainability. Data linking diet quality and sustainability are typically focused on a limited set of markers, and normally do not include food waste as an indicator (81, 82), despite a growing focus on understanding where and how food is wasted in the food system (83). Globally, enough food is wasted every year to feed nearly 2 billion people a 2,100 kcal/day diet (81), which magnifies the negative environmental impact associated with agriculture and resource scarcity. Food waste is a useful indicator of sustainability as it embodies all the resources used to produce uneaten food (83).

Recently it has been observed that some strategies to prevent food waste have been implemented in households inspired by growing social awareness of the matter (84). In the present study, it was found that women seemed more likely than men to use strategies related to household food management when trying to avoid food waste at home. This may be directly related to socially constructed gender roles, which give women greater responsibility for reproductive activities in the domestic environment, mainly related to food management (51, 85). Among the affiliations, teaching staff and administrative staff were more likely to employ actions related to food purchasing, such as planning shopping and meals and buying smaller quantities of food, compared to the students.

### Limitations

Despite the originality and relevance of this research, especially in Spain, some methodological limitations should be emphasized. First, the study was carried out within a single academic institution. Although the UB is one of the largest universities in Spain, the sample should be expanded to include other institutions and geographical contexts. Likewise, the convenience sample of this research may involve some risk of bias because the participants may already present a certain profile or interests related to the topic. Moreover, due to the underrepresentation of students among participants of this study, in future research it could be necessary to use other recruitment strategies to improve the student’s participation, as they may be key agents for the transformation of the food system in the next few years. Furthermore, the study is based on a quantitative approach. Although this methodology is widely used for the study of food perceptions (14, 59, 86), qualitative data could provide more in-depth results and new insights (87).

## Conclusion

This study has shown that in general the level of knowledge held by the analyzed university community about the more technical aspects of food sustainability is low, especially among students. Likewise, although different aspects of food sustainability generate a high level of concern in the student population, especially in women, sustainability is not among the main factors that influence food decisions. Finally, regarding perceptions, a less than holistic conception of sustainability has been revealed that does not include the social and economic dimensions.

The direction of the UB is committed to the SDGs and is carrying out various actions to implement them in the different areas of interest and among all the university groups with the aim of promoting sustainability in the academic sphere. Overall, the results indicate that a greater effort is needed to enhance knowledge of food sustainability and to improve the importance given to this dimension in food choice in the university community. Moreover, the findings of the present study highlight that these strategies should be designed taking into account the differences between the different affiliations.

## Data availability statement

The original contributions presented in this study are included in the article/Supplementary material, further inquiries can be directed to the corresponding author.

## Author contributions

MG, RC-S, OC-B, ML-M, GL-C, MP-L, and MCV-C: conceptualization. MG, RC-S, OC-B, and ML-M: investigation and writing—original draft preparation. MG, RC-S, OC-B, ML-M, and MA: data analysis. MG, RC-S, OC-B, ML-M, MA, GL-C, MP-L, and MCV-C: writing—review and editing. GL-C, MP-L, and MCV-C: supervision. All authors have read and agreed to the published version of the manuscript.
