# Supplementary material for: Knowledge and perceptions of food sustainability in a Spanish university population
Source: Front Nutr. 2022 Nov 29;9:970923. doi: 10.3389/fnut.2022.970923 (PMC9745073; doi:10.3389/fnut.2022.970923)
Supplement: Supplementary file 1 [file Data_Sheet_1.PDF]

Food and Sustainability Questionnaire

1. I have read the study objectives and have decided to take part voluntarily
   - Yes
   - No

2. Gender
   - Woman
   - Man
   - Non-binary
   - I prefer not to answer
   - Other:

3. Age

4. University group to which you belong:
   - Teaching and research staff
   - Administrative and service staff
   - Students

5. Level of studies completed
   - Secondary, upper secondary or equivalent
   - Bachelor’s degree
   - Master’s degree
   - Doctoral degree

6. Faculty, centre or service to which you belong:
   - Faculty of Fine Arts
   - Faculty of Biology
   - Faculty of Earth Sciences
   - Faculty of Law
   - Faculty of Economics and Business
   - Faculty of Education
   - Faculty of Pharmacy and Food Sciences (Campus Torribera)
   - Faculty of Pharmacy and Food Sciences (Campus Diagonal)
   - Faculty of Philology and Communication
   - Faculty of Philosophy
   - Faculty of Physics
   - Faculty of Geography and History
   - Faculty of Information and Audiovisual Media
   - Faculty of Mathematics and Informatics
   - Faculty of Medicine and Health Sciences (Campus Clínic)
   - Faculty of Medicine and Health Sciences (Campus Bellvitge)
   - Faculty of Psychology
   - Faculty of Chemistry
   - General administrative units
   - Scientific, technical and other specialized services
   - UB Group
   - Affiliated centres
   - Other:

7. Monthly household income (optional)
   - Up to €499
   - From €500 to €999
   - From €1,000 to €1,499
   - From €1,500 to €1,999
   - From €2,000 to €2,499
   - From €2,500 to €2,999
   - From €3,000 to €4,999
   - Over 5,000

8. With what word do you associate the concept of "food"? (Write one word only)

9. What are the two aspects that most influence your food choices?

- Nutritional composition of foods
- Time and ease for preparation and consumption
- Price
- Concern about body weight and/or fitness
- Ecology, environment and/or animal welfare
- Place of origin of food and support for agroecological territory
- Seasonality (seasonal product)
- Pleasure and taste preferences
- Disease prevention/health effects
- Mood

10. Indicate your degree of concern for each of the following aspects relating to food and diet (1 not at all concerned and 5 very concerned)

	1	2	3	4	5
Pesticides					
Hygiene in the home and outside the home					
Contamination by viruses (avian flu, norovirus, SARS-CoV-2, etc.) and bacteria (salmonella, listeria, etc.)					
Allergens					
Presence of gluten and/or lactose					
Residues of antibiotics and hormones in animal products					
Presence of chemical contaminants (e.g. mercury, dioxins, microplastics and nitrosamines)					
Animal welfare					
Genetically modified organisms (transgenic)					
Chronic non-communicable diseases related to diet (diabetes, cardiovascular diseases, cancer, etc.)					
Weight gain					
Sugar and salt content					
Fat and saturated fat content					
Food additives (colorants, preservatives, etc.)					
Food waste					
Use of plastics and plastic packaging					
Environmental impact (carbon footprint, water footprint, etc.)					
Socioeconomic situation of local agriculture					

11. To what extent do the items below make you confident about the quality of food? (1: no confidence; 5: total confidence)

	1	2	3	4	5
Brand					
Origin/place of production					
External appearance of the product					
Type of packaging					
Place of purchase					
Type of ingredients it contains					
Quality labels					
Price					
Type of manufacture or production					
Expiry/best before date					

12. How often have you heard about the environmental impact of food?
 - Often
 - Occasionally
 - Hardly ever
 - Never

13. Have you heard of the Sustainable Development Goals (SDGs) and the 2030 Agenda?
 - Yes
 - No

14. Have you heard of the European Green Deal?
 - Yes
 - No

15. How would you rate your knowledge of concepts relating to food sustainability and environmental sustainability? (1: know little; 5: know a lot)

	1	2	3	4	5
Carbon footprint					
Water footprint					
Biodiversity					
Local products (km 0)					
Greenhouse gases					
Food waste					

16. How important is food sustainability when you are buying products?
 - Extremely important
 - Quite important
 - Moderately important
 - Not very important
 - Not important

17. Would you say that you have a sustainable diet?
 - Yes, always
 - Yes, sometimes
 - No, never
 - I don’t know

18. Would you say that you have a healthy diet?
 - Yes, always
 - Yes, sometimes
 - No, never
 - I don’t know

19. Do you think that a healthy diet is the same as a sustainable diet?
 - Yes, always
 - Yes, sometimes
 - No, never

I don't know

20. What word do you associate with sustainable food? (Write one word only)

21. What word do you associate with healthy food? (Write one word only)

22. To what extent do you think the following aspects are related with sustainable food? (1: not related; 5: strongly related)

Environmental impact of agri-food systems
Affordable food for the entire population
Protection of biodiversity and ecosystems
Economically and socially fair methods of obtaining, producing and distributing foods
Safe, nutritious and healthy foods

23. A sustainable diet could include all the aspects below. In your opinion, which are the three most important aspects for a sustainable diet?

Affordable

Culturally acceptable

Rich in plant-based foods (flexitarian, vegetarian or vegan)

With locally produced, seasonal products

Organic

Simple, with no additives and based on foods with few ingredients that are not very processed

Respectful of ecosystem biodiversity and with a low environmental impact

With products from companies that respect workers' social rights

With no or the minimum amount of food waste

With biodegradable, compostable packaging

24. To what extent should the following foods/food groups form part of a sustainable diet? (1 is never and 5 is always)

	I don't know	1	2	3	4	5
Red meat						
White meat						
Meat derivatives						
Fish						
Dairy and derivatives						
Eggs						
Vegetables						
Potatoes and other root vegetables						
Fruit						
Nuts						
Refined grains						
Whole grains						
Pulses						
Olive oil						
Seed oil (sunflower, corn and flax)						
Snacks, sweets and pastries						
Sweetened beverages						
Coffee or tea						
Fermented alcoholic beverages (wine, beer)						
Distilled alcoholic beverages (gin, whisky)						

25. To what extent are the following aspects an impediment to a sustainable diet? (1: not an impediment; 5: a great impediment)

	1	2	3	4	5
Cost					
Lack of information					
Lack of culinary knowledge					
Lack of time					
Food preferences and taste					
Food customs and traditions					
Ease of purchase (accessibility)					

26. To what extent should the following foods/food groups be part of a healthy diet? (1 is never and 5 is always)

	I don't know	1	2	3	4	5
Red meat						
White meat						
Meat derivatives						
Fish						
Dairy and derivatives						
Eggs						
Vegetables						
Potatoes and other root vegetables						
Fruit						
Nuts						
Refined grains						
Whole grains						
Pulses						
Olive oil						
Seed oil (sunflower, corn and flax)						
Snacks, sweets and pastries						
Sweetened beverages						
Coffee or tea						
Fermented alcoholic beverages (wine, beer)						
Distilled alcoholic beverages (gin, whisky)						

27. How often do you think you waste food in your home?

Frequently
Occasionally
Hardly ever
Never

28. To what extent are the following actions important to avoid food waste in your home? (1 not important and 5 totally important)

	1	2	3	4	5
Use leftovers					
Plan shopping and meals					
Write shopping lists					
Buy smaller quantities of food					
Learn cooking techniques to preserve foods					
Make organic compost					
Consume foods that last longer (frozen foods, preserves)					
Take leftovers from a restaurant home					
Take part in initiatives to recover or make use of foods such as Too Good To Go					
